# Association between sexual violence and unintended pregnancy among adolescent girls and young women in South Africa

**DOI:** 10.1186/s12889-020-09488-6

**Published:** 2020-09-07

**Authors:** Anthony Idowu Ajayi, Henrietta Chinelo Ezegbe

**Affiliations:** 1 Population Dynamics and Sexual and Reproductive Health, Africa Population and Health Research Center, Off Kirawa Road, Manga Close, Nairobi, Kenya; 2 Faculty of Health Sciences, Simon Fraser University, Burnaby, British Columbia Canada

**Keywords:** Unintended pregnancy, Unplanned pregnancy, Survivors, Sexual violence, Contraception, Adolescent and young women, Abortions

## Abstract

**Background:**

Unintended pregnancy has dire consequences on the health and socioeconomic wellbeing of adolescent girls and young women (AGYW) (aged 15–24 years). While most studies tend to focus on lack of access to contraceptive information and services, and poverty as the main contributing factor to early-unintended pregnancies, the influence of sexual violence has received limited attention. Understanding the link between sexual violence and unintended pregnancy is critical towards developing a multifaceted intervention to reduce unintended pregnancies among AGYW in South Africa, a country with high teenage pregnancy rate. Thus, we estimated the magnitude of unintended pregnancy among AGYW and also examined the effect of sexual violence on unintended pregnancy.

**Methods:**

Our study adopted a cross-sectional design, and data were obtained from AGYW in a South African university between June and November 2018. A final sample of 451 girls aged 17–24 years, selected using stratified sampling, were included in the analysis. We used adjusted and unadjusted logistic regression analysis to examine the effect of sexual violence on unintended pregnancy.

**Results:**

The analysis shows that 41.9% of all respondents had experienced an unintended pregnancy, and 26.3% of those unintended pregnancies ended in abortions. Unintended pregnancy was higher among survivors of sexual violence (54.4%) compared to those who never experienced sexual abuse (34.3%). In the multivariable analysis, sexual violence was consistently and robustly associated with increased odds of having an unintended pregnancy (AOR:1.70; 95% CI: 1.08–2.68).

**Conclusion:**

Our study found a huge magnitude of unintended pregnancy among AGYW. Sexual violence is an important predictor of unintended pregnancy in this age cohort. Thus, addressing unintended pregnancies among AGYW in South Africa requires interventions that not only increase access to contraceptive information and services but also reduce sexual violence and cater for survivors.

## Background

Unintended pregnancy, especially among adolescent girls and young women (AGYW) remains a concerning health and social problem in sub-Saharan Africa (SSA) and worldwide [1, 2]. Between 2010 and 2014, approximately 44.5% of all pregnancies and 23% of births were unintended worldwide [2]. Unintended pregnancy is the main reason for having an abortion [3]. Of the approximately 99 million unintended pregnancies that occur each year, more than half (56%) end in abortion [2]. Unintended pregnancy has deleterious consequences, including a heightened risk of maternal depression [4], and late initiation of antenatal care [5].

Adolescent girls and young women (aged 15–24 years) are at high risk of having an unintended pregnancy because they are far more likely to enter into sexual relationships and also more likely to delay marriage in order to complete school and become better prepared to join the labour force [6]. A study conducted in Uganda shows that sexually active adolescent girls had the highest rate of abortion (76.1 abortions per 1000 women 15–19) of all women within the reproductive age group [7]. Another study in Kenya shows that severe complications of unsafe abortion were most common among adolescent girls [8].

With figures as high as 102.8 pregnant adolescents per 1000 girls aged 15–19 years, SSA has the highest rates of teenage pregnancies in the world [9]. Early unintended pregnancy has dire health and socioeconomic consequences. Globally, pregnancy and childbirth complications are one of the leading causes of death among adolescent girls. Adolescent mothers face an increased risk of perinatal morbidities such as eclampsia, puerperal endometritis, systemic infections, low birth weight, preterm delivery, and severe neonatal conditions [10–12]. Indeed, early adolescent pregnancy has been linked with an increased risk of HIV infection [13]. Furthermore, early childbearing also negatively impacts girls’ socioeconomic development and empowerment, further bolstering the vicious cycle of poverty [14–17].

In South Africa, AGYW continue to get pregnant at alarmingly high rates, with about 28% of girls having begun childbearing by their nineteenth birthday [18]. Contributing factors have been described as multifaceted, and include individual, household, community, and structural factors [19]. At the individual level, the high prevalence of unintended pregnancies has been associated with limited contraceptive knowledge, incorrect and inconsistent contraceptive use, inaccessibility of contraceptives [1, 20–22]. At the household and family level, household poverty, lack of family support, death of parents, lack of communication with parents about sex encounters, and single-parent family structure are associated with early-unintended pregnancies [23–27]. At the community level, community-level poverty, rural residence, cultural norms that foster gender inequality and violence against women are among the factors associated with a higher likelihood of unintended pregnancies [23, 24]. Structural factors associated with the increased level of early unintended pregnancy include restrictive policies - such as laws on age of consent for contraception services, healthcare systems failures, inadequate access to contraception as well as epileptic availability and access to reproductive healthcare services [28, 29].

While several studies have focused on the influence of contraceptive access and poverty on the increased risk of having an unplanned pregnancy [1, 20, 23], the contribution of sexual violence to increased risk of unintended pregnancy particularly among AGYW has received limited attention, with only a few studies from other regions focusing on this link [30–33]. The pathway through which sexual violence could lead to unintended pregnancy is through non-use of contraceptive, underreporting of incidences of sexual violence, and lack of requisite care to address the potential impacts of sexual violence, including unintended pregnancy [34, 35]. Individuals who rape young people are likely not to use condoms, and since the knowledge of emergency contraception is low among AGYW [36, 37], the risk of them having an unintended pregnancy is high. Also, given that victims of sexual violence rarely come forward, the risk of having unintended pregnancy is high as young people are rarely going to know how to prevent pregnancy after exposure to forced and coerced sex.

Several studies have linked sexual violence to increased risk of HIV infection [38, 39], poor mental health outcomes [40, 41], poor self-rated health [41] and non-use of contraceptives [39] among AGYW in South Africa, but sparse attention has been given to the effect of sexual violence and unintended pregnancy. However, studies have examined the link between coerced first sex and unintended pregnancy, reporting contrasting results [13, 42]. Christofides et al. (2014) show that physical abuse is associated with a higher likelihood of unintended pregnancy. But, they found no relationship between coerced first sex and unintended pregnancy in contrast to an earlier study by Maharaj and Munthree (2007). Overall, most studies on unintended pregnancy among AGYW in South Africa focused on the role of gender inequality, socioeconomic inequalities, and lack of contraceptive access and use, with limited attention to the role of sexual violence. As such, the main research question this study examines is: what is the link between sexual violence and unintended pregnancy among AGYW? Our study adds to the growing body of knowledge by determining the magnitude of unintended pregnancies as well as examining the relationship between sexual violence and unintended pregnancies among AGYW in South Africa. The study finding will be useful in developing policies and strategies to improve the sexual and reproductive health outcomes of young people. Specifically, our findings could inform the implementation of youth-friendly sexual and reproductive health services in South Africa.

## Methods

### Study design and setting

The cross-sectional study was conducted among students aged 17–24 years in a South African university located in Eastern Cape province. We selected the university conveniently in a setting with high sexual violence and HIV prevalence [43]. Our focus on AGYW is influenced by the fact that new HIV infection rates were disproportionately high among this cohort in South Africa [44]. University students were recruited because of ease of accessibility and lack of funds for a household survey. The full details of the methodology for the study have been published elsewhere [45, 46]. Only unmarried male and female students aged 15 to 24 years were eligible for the study. Visiting students from another university, married students, and those aged over 24 years were ineligible for selection.

A total of 420 participants were estimated to be the appropriate sample size for this study based on ±5% precision level, a 95% confidence level, and female students’ population of 4000 and 10% possible attrition, using MaCorr Sample Size Calculator. We employed stratified sampling to ensure representativeness. Stratification was based on the following characteristics of students, faculties, and years of study. We drew participants from all faculties based on probability proportionate to the size of the faculty, including social sciences and humanities (*n* = 138), law (*n* = 70), health sciences (55), management and commerce (92) and education (155). Students were also stratified by year of study to ensure the final sample is representative of the distribution of study by year of study. We recruited participants from their lecture halls because we were unable to get a comprehensive list of all students.

The study instrument was self-designed and included in Supplementary file 1. Well-trained research assistants administered the survey using ODK collect installed on android devices. The research assistants were postgraduate students and were trained on using the ODK collect application, ethical considerations guiding the research, and participants’ selection. We trained them to select a pre-specified number of students at a particular level and faculty of study. They were instructed to select every tenth student in the classroom and skip participants who refuse to participate. The ODK collect application ensured privacy. Participants were approached and informed about the study purpose. To minimise social desirability bias and further ensure privacy, consenting students completed the survey using either their personal mobile phones or the research assistants’ mobile device in private spaces earmarked for the study on campus. No personal identifying information was collected, and they were shown how to operate the ODK App for android and navigate the survey questions. The study was conducted between June and November 2018. We conducted training of research assistants and pilot testing of study instruments among 30 students using a different university before the study commenced. The response rate was 88% among the female participants included in this study.

The University of Fort Hare ethical review body approved this study (Reference number: GON011). All participants gave written consent indicating that they voluntarily and willingly took part in the study and affirmed that they understood the study purpose, process, and usage of findings. Anonymity and confidentiality of the information provided were ensured throughout the study. We followed all the IRB guidelines for using human subjects in research.

### Measures

Our dependent variable is the lifetime experience of unintended pregnancy, which we defined as becoming pregnant at a time a person is not prepared to become pregnant or intended to have children. We measured this by asking participants if they have ever been pregnant when they never wanted to get pregnant. We used a binary response of “yes” or “no” to categorise participants’ responses. We followed the question up by asking what action was taken when they found that they were pregnant. The actions were classified as terminated the pregnancy and carried the pregnancy to term.

Our main independent variable of interest is sexual violence. Sexual violence is defined in this study as any sexual act or attempts to obtain a sexual act by violence or coercion by any person irrespective of their relationship to the victim [47]. We asked respondents if they have ever experienced sexual abuse, such as forced sex or rape and touching of genitals without proper consent. We favoured a narrow definition of sexual violence in this study by excluding verbal sexual assault, which may not lead to unintended pregnancy. Also, we asked if they experienced this sexual abuse before the age of sixteen. The responses were classified as yes or no.

We included three sets of covariates, individual level, behavioural and household, and family level covariates based on existing literature [48–51]. The individual-level covariates include age, religion, and parity. Age was measured as a continuous variable by asking respondents to state their age at their last birthday. We later categorised the ages into late-adolescent girls (17–19 years) and young women (20–24 years). Religiosity was measured by asking participants to rate their level of religiosity out of ten. We classified those who rated themselves 8 to 10 as very religious, those who rated themselves from 5 to 7 as moderately religious, and 1 to 4 as not religious. Lastly, we asked respondents to state how many children they have ever had.

The behavioural factors include recreational drug use and relationship status. We asked participants to indicate if they ever used drugs such as dagga, codeine, cannabis, and/or tramadol for pleasure or to ease tension or stress. Also, we asked respondents if they are single or cohabiting. We classified their responses as yes or no. We included several family/household level factors, including family structure, family support, death of parents, communication of sexual encounters with parents, and parenting type. Family structure was classified as single-parent, polygamous, both parents, and foster family.

Family support was used as a proxy to measure parents’ socioeconomic status. We asked participants to rate the level of support they received from their family as adequate, moderate, insufficient, and no support. Family support relates to financial support and not psychological support. Also, we measured communication with parents regarding sexual encounters by asking respondents if they have ever discussed sex with any or both of their parents. Lastly, we measured parenting style by asking respondents to describe their parents as strict and not strict.

### Statistical analysis

The analytical sample of the current paper begins with 510 AGYW, who took part in the survey. From this number, we remove 59 respondents who refused to answer the questions on sexual behaviours, unintended pregnancy, sexual violence, and HIV testing. These sets of participants exercised their right to refuse to answer any questions they feel uncomfortable answering and to drop out of the study at any time. We examined the characteristics of these sets of respondents and did not find any significant difference between them and those included in our analysis. We, therefore, analysed data from 451 AGYW who returned with complete responses representing a response rate of 88.4% among female participants. We performed descriptive statistics of all variables included in this study. We present mean and standard deviation for age. To determine whether sexual violence was associated with a higher likelihood of unintended pregnancy, we fitted two logistic regression models. The first model was a baseline model with no covariates, which was used to estimate the unadjusted odds ratio of the association between the main independent variable and the dependent variables. The second model is a multivariable model, where we included all relevant covariates, including individual, behavioural and family, and household level factors. Alpha level of less than 0.05 was considered to be statistically significant.

## Results

### Descriptive findings

The average age of respondents was 21.03 (SD: 1.61) years. As shown in Table 1, most respondents were aged 20–24 years (79.6%), single (97.8%), had no children (65.4%), and never drank alcohol (66.5%). Only 38.8% were from a two-parent family. While a majority live their mother (75.4%), only two-fifth of them live with their father. Only a few respondents described their parents as strict (father 30.8%, mother 26.4%). While most of the respondents have discussed sex with their mother (61.6%), only a few of them have talked about sex with their father (10.4%). Over a third of the respondents (37.9%) have ever experienced sexual violence, and 17.7% did before the age of 16.

**Table 1:** Socio-demographic, behavioural and household characteristics of respondents

Variable	Frequency	Percentages
Age		
17–19	92	20.4
20–24	359	79.6
Relationship status		
Single	441	97.8
Cohabiting	10	2.2
Parity		
None	295	65.4
One	150	33.3
More than one	6	1.3
Family support		
Adequate	168	37.3
Moderate	202	44.8
Insufficient support	81	18.0
Religiosity rating		
Not religious	139	32.0
Moderately religious	197	45.4
Very religious	98	22.6
Family structure		
Two parents family	175	38.8
Single parent family	196	43.5
Living with grandparents	49	10.9
Living with foster parents	31	6.9
Mother alive	377	83.6
Father alive	282	62.5
Live with dad	190	42.1
Live with mum	340	75.4
Talk sex to dad	47	10.4
Talk sex to mum	278	61.6
Strict father	139	30.8
Strict mother	119	26.4
Ever drank alcohol	300	66.5
Current alcohol users	201	44.6
Drank alcohol last week	108	23.9
Ever used drugs	138	30.6
Currently use drugs	51	11.3
Lifetime experience of sexual abuse	171	37.9
Past year experience of sexual abuse	114	25.3
Childhood sexual abuse	80	17.7

**Fig. 1:**
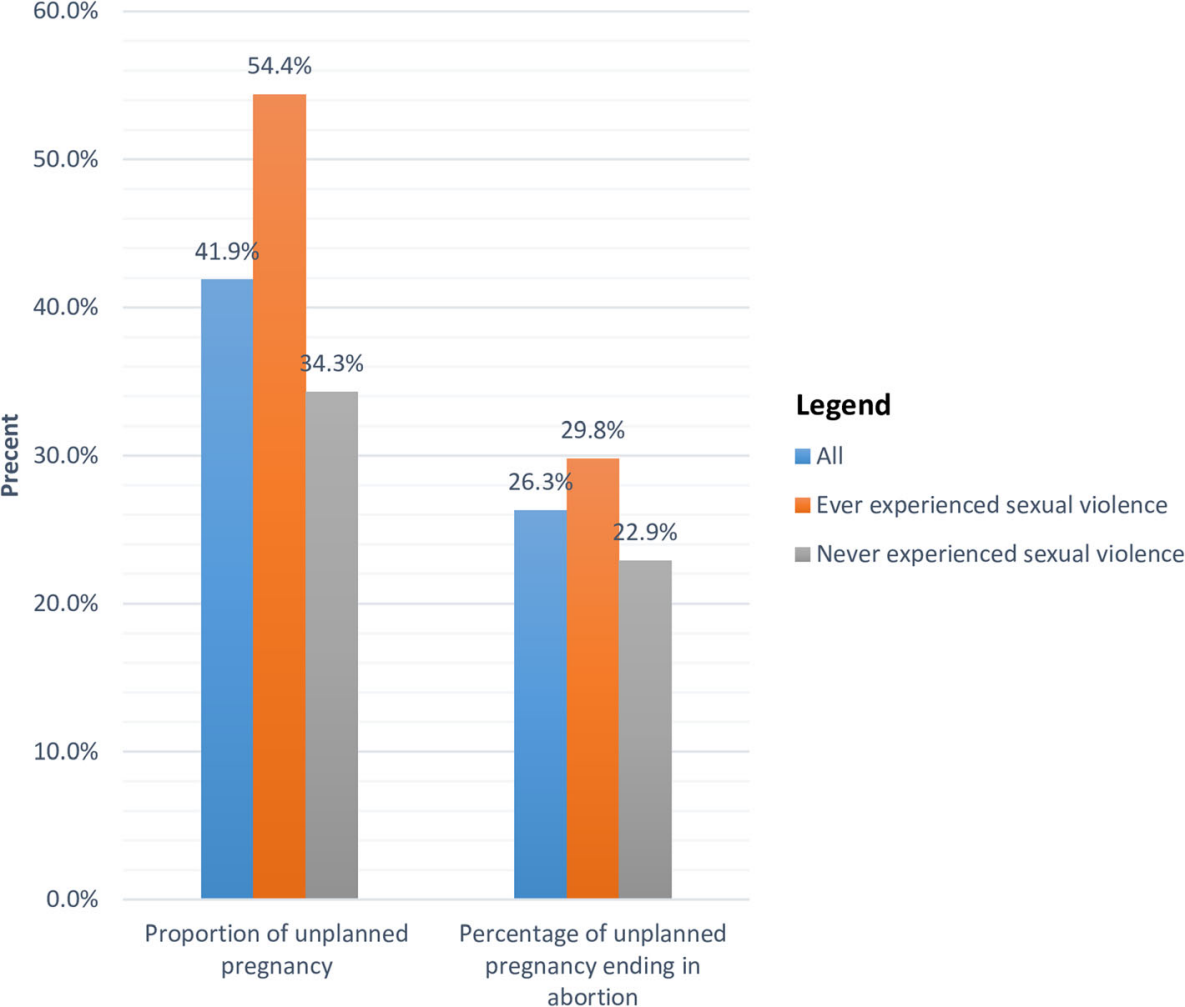
Unintended pregnancy and related abortion among adolescent and young women

As presented in Fig. 1, 41.9% of all respondents had experienced an unintended pregnancy, and 26.3% of those unintended pregnancies ended in abortions. Unintended pregnancy was higher among young women (20–24 years) (47.1%), those who received no or insufficient support (58.0%), those who rated themselves not religious (54.7%), those who had ever consumed alcohol (48%), and survival of sexual violence (54.4%) compared to adolescent girls (21.7%), those who received adequate support (33.9%), those that rated themselves as very religious (30.6%), those who never drank alcohol (29.8%), and those never experienced sexual assault (34.3%) respectively (Table 2).

**Table 2:** Chi-square statistics showing factors associated with having had an unintended pregnancy

Variables	Never had an unintended pregnancy	Ever had an unintended pregnancy	*P*-value
Lifetime experience of sexual abuse			
Yes	78 (45.6)	93 (54.4)	< 0.001
No	184 (65.7)	96 (34.3)	
Age			
17–19	72 (78.3)	20 (21.7)	< 0.001
20–24	190 (52.9)	169 (47.1)	
Family support			
Adequate	111 (66.1)	57 (33.9)	0.001
Moderate	117 (57.9)	85 (42.1)	
No or insufficient support	34 (42.0)	47 (58.0)	
Family structure			
Two parents family	106 (54.1)	90 (45.9)	0.304
Single parent family	109 (62.3)	66 (37.7)	
Living with grand parents	31 (63.3)	18 (36.7)	
Living with foster parents	16 (51.6)	15 (48.4)	
Religiosity rating			
Not religious	63 (45.3)	76 (54.7)	< 0.001
Moderately religious	123 (62.4)	74 (37.6)	
Very religious	68 (69.4)	30 (30.6)	
Parity			
None	248 (84.1)	47 (15.9)	< 0.001
At least one	14 (9.0)	142 (91.0)	
Ever drank alcohol			
Yes	156 (52.0)	144 (48.0)	< 0.001
No	106 (70.2)	45 (29.8)	
Ever used drugs for recreational purposes			
Yes	70 (50.7)	68 (49.3)	0.023
No	192 (61.3)	121 (38.7)	
Life time experience of sexual abuse			
Yes	49 (43.0)	65 (57.0)	< 0.001
No	213 (63.2)	124 (36.8)	
Strict father			
Yes	81 (68.1)	38 (31.9)	0.007
No	181 (54.5)	151 (45.5)	
Strict mother			
Yes	91 (65.5)	48 (34.5)	0.021
No	171 (54.8)	141 (45.2)	
Sex talk with father			
Yes	35 (74.5)	12 (25.5)	0.011
No	227 (56.2)	177 (43.8)	
Sex talk with mother			
Yes	153 (55.0)	125 (45.0)	0.058
No	109 (63.0)	64 (37.0)	

### Multivariable findings

As shown in Model 1, sexual violence was associated with higher odds of having an unintended pregnancy (Table 3). In Model 2, we included individual, behavioural and family, and household-level covariates. After adjusting for all covariates, the magnitude of the effect reduced, but the direction of effect persists, showing that when adolescents and young adults reported having experienced sexual violence, they are more likely to have also had an unintended pregnancy. Alcohol use and increasing age were also significantly associated with a lifetime experience of unintended pregnancy. However, family structure, family support, religiosity, and parenting style were not significantly associated with unintended pregnancy.

**Table 3:** Logistic regression models of the association between sexual violence and unintended pregnancy

Variables	Unadjusted model	Adjusted model
Life experience of sexual abuse		
Yes	2.29 (1.55–3.37)***	1.70 (1.08–2.68)*
No	1	1
Age		
20–24		3.29 (1.83–5.93)***
17–19		1
Family support		
Adequate		0.71 (0.39–1.30)
Moderate		0.65 (0.34–1.24)
No or insufficient support		1
Family structure		
Two parents family		0.92 (0.36–2.37)
Single parent family		0.89 (0.33–2.40)
Living with grand parents		0.79 (0.36–2.37)
Living with foster parents		1
Religiosity rating		
Poor		1.76 (0.96–3.23)
Average		1.09 (0.62–1.90)
Good		1
Ever drank alcohol		
Yes		1.68 (1.03–2.73)*
No		1
Ever used drugs for recreational purposes		
Yes		0.94 (0.58–1.54)
No		1
Strict father		
Yes		0.64 (0.37–1.10)
No		1
Strict mother		
Yes		0.66 (0.41–1.04)
No		1
Sex talk with father		
Yes		0.68 (0.32–1.45)
No		1
Sex talk with mother		
Yes		1.26 (0.75–2.12)
No		1

*pvalues <0.05 ***pvalues <0.001

## Discussion of findings

This study examines the relationship between sexual violence and unintended pregnancies among AGYW in South Africa. We found a high prevalence of unintended pregnancy (41.5%) among AGYW in our study setting, with a quarter of all unintended pregnancies resulting in abortions. Over 91% of pregnancies among AGYW are unintended in our study cohort. Our findings further add to the growing body of knowledge, showing a high prevalence of unintended pregnancy among AGYW in South Africa. Underuse of contraceptives has been attributed to the high rate of unintended pregnancy among AGYW in SSA [20]. Thus, addressing unintended pregnancy among AGYW in South Africa will require increasing access to contraceptive information and services.

Our result, consistent with previous studies [30–33], demonstrates clear, consistent, and robust evidence supporting our proposition that AGYW with lived experiences of sexual violence (AOR:1.76, 95% CI:1.07–2.90) are more likely to report unintended pregnancies. The pathway through which sexual violence could lead to unintended pregnancy is through non-use of contraceptive, underreporting of incidences of sexual violence, and lack of requisite care to address the potential impacts of sexual violence, including unintended pregnancy. It is clear from extant studies that most victims of sexual violence do not officially report rape incidence or even mention it to other people [34]. Lack of reporting of sexual violence incidences may mean survivors will not receive the necessary care needed. Also, it is well established that most perpetrators of sexual violence are close to the survivors, with friends and boyfriends being the most likely culprits, making the incidence more frequent and the consequences severe [35]. For example, the prevalence of forced sexual initiation ranges from 4 to 31% [40]. AGYW are also at a disadvantage because they have low knowledge of after sex contraception [36, 37], implying that if they fail to report or seek care, their risk of unintended pregnancy increases.

Given that early-unintended pregnancies worsen the socioeconomic outcomes of AGYW [14–17], there is a need for research that examines how sexual violence against women could worsen gender inequalities and female poverty. Aside from providing access to contraceptive information and services, there is a need for holistic efforts to expand young people’s knowledge and awareness around female sexual and reproductive health rights. Furthermore, it is vital that sex education, which highlights the damaging effects of sexual violence, discourages hegemonic masculinity, and encourages responsible sexual decision making, be included in schools’ curriculums, and be made an integral part of religious/community body events to ensure that out-of-school children are not left out. Parents, caregivers, and guardians should additionally assume the responsibilities of teaching these important lessons at home. As social pressure or fear of stigma may mean that sexual violence cases are underreported, it is crucial that responsible authorities launch appropriate investigations when cases of sexual assaults are reported, and ensure justice served accordingly in addition to providing judgment-free victim support, which will encourage other victims to speak up.

### Strength and limitations

Our study provides needed evidence on the link between sexual violence and unintended, which could provide the basis for future studies and intervention to tackle sexual violence and unintended pregnancy among AGYW in South Africa and beyond. However, our study is not without limitations. The use of cross-sectional data means causal inference could not be drawn between sexual violence and unintended pregnancy. Also, underreporting of unintended pregnancy and sexual violence could not be ruled out given the sensitive nature of the topic. However, the use of ODK collect for android offers privacy for participants, thereby limiting the effect of social desirability bias. Also, our use of secondary dataset means that our study did not include all potential confounding factors, including knowledge of emergency contraception and access to emergency contraception. Lastly, our focus on university students may not adequately portray a real-world situation for the target age bracket, and thus limits our study’s generalizability. University students are generally more educated compared with the general population of AGYW. Also, our study is limited to only unmarried AGYW; as such, the findings are not generalizable to married AGYW.

## Conclusions

Consistent with previous research in sub-Saharan Africa, our study found a high prevalence of unintended pregnancy among adolescents and young women, suggesting that despite improved access to contraceptives in South Africa, the magnitude of unintended pregnancy remains high. We also found that sexual violence is associated with a higher likelihood of unintended pregnancy. As such, addressing unintended pregnancies among AGYW in South Africa requires interventions that not only increase access to contraceptive information and services but also reduce sexual violence and cater for survivors. Future studies should examine the link between sexual violence and unintended pregnancy among out of school girls as well as the potential link between exposure to sexual violence and gender inequality and female poverty.

## Supplementary information

**Additional file 1.** Study questionnaire.

## Data Availability

The data analysed will be made available by the corresponding author upon request.

## Abbreviations

AGYW: Adolescent girls and young women
SSA: Sub-Saharan Africa
HIV: Human immunodeficiency virus
